# Bioconversion to Raspberry Ketone is Achieved by Several Non-related Plant Cell Cultures

**DOI:** 10.3389/fpls.2015.01035

**Published:** 2015-11-24

**Authors:** Suvi T. Häkkinen, Tuulikki Seppänen-Laakso, Kirsi-Marja Oksman-Caldentey, Heiko Rischer

**Affiliations:** ^1^VTT Technical Research Centre of Finland Ltd.Espoo, Finland

**Keywords:** betuligenol, bioconversion, 4-hydroxybenzalacetone, plant cell culture, raspberry ketone

## Abstract

Bioconversion, i.e., the use of biological systems to perform chemical changes in synthetic or natural compounds in mild conditions, is an attractive tool for the production of novel active or high-value compounds. Plant cells exhibit a vast biochemical potential, being able to transform a range of substances, including pharmaceutical ingredients and industrial by-products, via enzymatic processes. The use of plant cell cultures offers possibilities for contained and optimized production processes which can be applied in industrial scale. Raspberry ketone [4-(4-hydroxyphenyl)butan-2-one] is among the most interesting natural flavor compounds, due to its high demand and significant market value. The biosynthesis of this industrially relevant flavor compound is relatively well characterized, involving the condensation of 4-coumaryl-CoA and malonyl-CoA by Type III polyketide synthase to form a diketide, and the subsequent reduction catalyzed by an NADPH-dependent reductase. Raspberry ketone has been successfully produced by bioconversion using different hosts and precursors to establish more efficient and economical processes. In this work, we studied the effect of overexpressed *Ri*ZS1 in tobacco on precursor bioconversion to raspberry ketone. In addition, various wild type plant cell cultures were studied for their capacity to carry out the bioconversion to raspberry ketone using either 4-hydroxybenzalacetone or betuligenol as a substrate. Apparently plant cells possess rather widely distributed reductase activity capable of performing the bioconversion to raspberry ketone using cheap and readily available precursors.

## Introduction

The characteristic aroma component in raspberry (*Rubus idaeus*) fruits is 4-(4-hydroxyphenyl)butan-2-one, also called raspberry ketone or frambinone (Feron et al., 2007). The amount of raspberry ketone in raspberry fruits is only around 1–4 mg/kg fruits. Raspberry ketone is one of the most expensive flavor compounds. Natural raspberry ketone flavor ranks second behind natural vanillin, with a total potential market value between 6 and 10 million euros (Feron and Wache, 2005), although currently the commercial demand cannot be satisfied. The EC Flavor Directive (88/388/EEC) defines natural flavors as ‘flavoring substances or preparations which are obtained by appropriate physical processes or enzymatic or microbiological processes from material of vegetal or animal origin.’ Natural flavors include products obtained through microbial or enzymatic processes as long as the precursor/raw material is natural and obtained via physical or bio-processes and the precursor and product can be found in nature or as components of traditional foods. Products that occur in nature but are produced via a chemical (non-biological) process are called ‘nature-identical’; this mode of production is no longer accepted as consumer-friendly (Vandamme and Soetaert, 2007). In addition to traditional flavoring applications, raspberry ketone has attracted wide interest in the so-called cosmaceutical industry, for its skin-lightening and weight-loss properties (Morimoto et al., 2005; Park, 2010; Lin et al., 2011).

The biosynthesis of this industrially sought-after flavor compound is a comparatively well characterized diketide pathway, involving the condensation of 4-coumaryl-CoA and malonyl-CoA. First coumaroyl-CoA and malonyl-CoA form *p*-hydroxybenzalacetone (4-OHBA) in a decarboxylative condensation catalyzed by a Type III polyketide synthase called benzalacetone synthase (BAS) (Abe et al., 2001). Several candidate polyketide synthases from *Rubus* have been identified (*Ri*PKS1-5), and among those *Ri*PKS4 exhibits a specific C-terminal sequence which is different from the usually conserved region in chalcone synthases (CHSs) (Zheng et al., 2001; Zheng and Hrazdina, 2008). *Ri*PKS4 has both BAS and CHS activity *in vitro*, and therefore selective blocking of the CHS activity of *Ri*PKS4 would be difficult to achieve. Instead it seems very likely that the reaction toward benzalacetone (and further to raspberry ketone) is determined by precursor availability and especially the interaction with a specific reductase. Koeduka et al. (2011) identified an NADPH-dependent reductase from raspberry, called raspberry ketone/zingerone synthase 1 (*Ri*ZS1), which was suggested to be responsible for the last step in raspberry ketone biosynthesis. However, this gene has not hitherto been functionally tested *in planta*.

**FIGURE 1:**
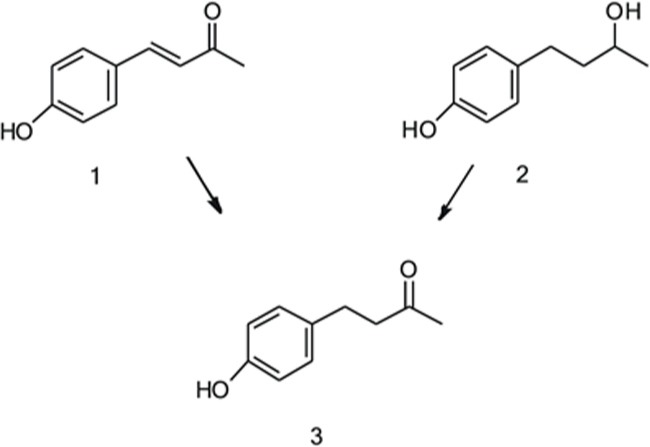
**Conversion of 4-hydroxybenzalacetone (1) and betuligenol (2) into raspberry ketone (**3**)**.

Plant cell cultures have been studied as useful agents for various biotransformation reactions of organic compounds, including oxidation, reduction, hydroxylation, esterification, methylation, isomerization, hydrolysis and glycosylation (Giri et al., 2001; Ishihara et al., 2003). Raspberry ketone has been produced via bioconversion with different hosts including bacteria, yeast and plant cells, using e.g., *p*-coumaric acid, benzoic acid, benzaldehyde, 4-OHBA (1) or betuligenol (or rhododendrol) (2) as a precursor (**Figure 1**) (Fischer et al., 2001; Beekwilder et al., 2007).

The berry-derived precursor **1** is not abundant in nature, but it can be produced by bacterial cultivation via condensation of hydroxybenzaldehyde and acetone (Feron et al., 2007). **2** is a secondary alcohol isolated originally from *Taxus wallichiana* (Chattopadhyay et al., 2001). It is also found, in e.g., birch bark, rhododendron, alder, maple and fir, mainly in its glycosylated form called betuloside. Betuloside can be converted into betuligenol, by e.g., microbial β-glucosidase (Dumont et al., 1996). Bioconversion of **2** into **3** has been successfully achieved by various microbial cells (Dumont et al., 1996; Kosjek et al., 2003) and *Atropa belladonna* hairy roots (Srivastava et al., 2013). In a study by Kosjek et al. (2003), the oxidation reaction from **2** to **3** was performed in the actinomycete *Rhodococcus* by using acetone as a hydrogen acceptor. However, when *A. belladonna* hairy roots were fed with **2**, both **3** and betuloside were formed without the requirement of an additional co-substrate. On the other hand, Fujita et al. (1998) showed that callus cultures of *Acer nikoence* (Nikko maple) converted fed **3** to **2** and their glycosides. Interestingly, **3** was only found in the culture medium, whereas glycosides were present in the intracellular space. The authors suggested that a certain specific alcohol dehydrogenase (ADH) and a glycosyltransferase participate in these reactions. Bioconversion of **1** was accomplished with various microbial cells, yielding raspberry ketone with varying conversion efficiencies (Fuganti and Zucchi, 1998). They observed that with longer incubation times, conversion of **1**–**3** continued to formation of **2**. It is known that a reaction from **1** to **3** is catalyzed by an NADPH-dependent enzyme, which was characterized from *Rubus idaeus* by Koeduka et al. (2011). On the other hand, the conversion step from **2** to **3** has not yet been characterized. Indeed, Beekwilder et al. (2007) showed that *Escherichia coli* possesses an endogenous reductase activity to convert **1** into **3**, after *p*-coumaric acid feeding to the cells expressing BAS. It is thus likely that the reductase activity required for this conversion is rather widely distributed in nature. In this study we present bioconversion studies related to raspberry ketone, performed with various plant cell cultures derived from plant species unrelated to raspberry.

## Materials and Methods

### Plant Material and Precursors

Hairy roots of *Nicotiana tabacum* SR1 ‘Petite Havana’ (tobacco, VTT Culture Collection no. VTTCC P-120068, **>Supplementary Figure S3**) and *Catharanthus roseus* (Madagascar periwinkle, VTTCC P-120070) as well as *N. tabacum* cell suspension cultures SR1 (VTTCC P-120003) and BY-2 (VTTCC P-120001) were initiated and maintained as described by Häkkinen et al. (2012). Hairy roots of *Hyoscyamus muticus* (Egyptian henbane, VTTCC P-120039, **Supplementary Figure S4**) were initiated and maintained as described in Häkkinen et al. (2005). *Hordeum vulgare* ‘Pokko’ cell suspension culture (barley, VTTCC P-120080) was established and maintained as described in Ritala et al. (1993). *Rubus idaeus* (raspberry, VTTCC P-120090), *Rubus chamaemorus* (cloudberry, VTTCC P-120083, **Supplementary Figure S2**) and *Rubus arcticus* (arctic bramble, VTTCC P120089) were maintained as described in Nohynek et al. (2014) with slight modifications (**Supplementary Figure S5**). *Vaccinium myrtillus* (bilberry, VTTCC P-120045) cell suspension was maintained in modified McCown Woody Plant medium (McCown and Lloyd, 1981). *Plumbago auriculata* (leadwort, VTTCC P-120006) cell suspension culture was maintained in modified Murashige and Skoog’s medium (Murashige and Skoog, 1962). *N. benthamiana* was cultivated according to Joensuu et al. (2010). Precursors *p*-hydroxybenzalacetone (4-OHBA) and betuligenol (rhododendrol) were purchased from Sigma–Aldrich (S350656, USA) and TCI America (R0121, USA), respectively. Raspberry ketone was obtained from Sigma–Aldrich (W258806, USA).

### *Ri*ZS1 Plant Vector Construct

*Rubus idaeus* ketone/zingerone synthase 1 (*Ri*ZS1) with NCBI Accession no. JN166691.1 was ordered from GenScript USA Inc. (USA) and cloned into the Gateway^®^ plant compatible vector pK2GW7 (Life Technologies^TM^) according to manufacturer instructions. The resulting vector carrying 35*S*-*Ri*ZS1 was transformed into *Agrobacterium rhizogenes* LBA9402 and *A. tumefaciens* LBA4404 by electroporation. The presence of the transgene was confirmed by PCR using Gateway^®^ primers 5′-GGGGACAAGTTTGTACAAAAAAGCAGGC-3′ and 3′-GGGGACCACTTTGTACAAGAAAGCTGGG-5′ for amplification of the vector region between ATTB1 and ATTB2 sites.

### Transient Expression in *N. benthamiana*

The *Agrobacterium* suspensions were infiltrated into the leaves of 6-week-old *N. benthamiana* plants as described previously (Joensuu et al., 2010). Briefly, *N. benthamiana* plants were cultivated in a greenhouse for 6 weeks before infiltration. One day prior to infiltration a bacterial suspension was inoculated and incubated at +28°C overnight. The cultures were diluted to OD_600_ 0.35 with 10 mM MgSO_4_, 10 mM MES buffer. Buffer-infiltrated leaves were used as control. LBA4404 carrying p19 silencing suppressor was tested for enhanced *Ri*ZS1 expression. Bacterial suspensions carrying p19 and *Ri*ZS1 were applied together by mixing the suspensions either by adding both in equal amounts (1:1 v:v) or adding one fifth of p19 (1:4 v:v). Each infiltration culture was applied to five different leaves at 1 ml total volume per leaf. After infiltration, the leaves were blotted dry with paper tissue and the plants were transferred back to the greenhouse. Sampling was performed after incubation for 6 days. Altogether 20 leaf disks (15 mm aaa) per sample were collected and placed on a petri dish. Substrate (1 mM 4-OHBA) was diluted in 20 mM phosphate-citrate buffer (pH 7.4), and petri dishes were subjected to vacuum conditions twice. After 2 days of incubation, the samples were frozen in liquid nitrogen and stored at -80°C until analyses. Each sample was taken in triplicate.

### Biotransformation

Cell suspension cultures were inoculated for 5 days and hairy roots for 9 days prior to feeding, as described in Häkkinen et al. (2012). Precursor was diluted in sterile water and was fed at a final concentration of 100, 300, or 500 μM. Samples were collected after 1, 2, or 5 days and medium was separated from cells by filtering. Cell and medium samples were frozen and lyophilized before extraction.

### Sample Extraction and GC-MS Analyses

Plant material was grinded with a Retsch mill (MM301, GWB, Germany) into a fine powder. Samples were weighed (50 mg lyophilized material) and 2 ml ultra-pure water was added. Alternatively, 3 ml medium samples were taken for the extraction and spiked with internal standard (20 μg heptadecanoic acid). Raspberry ketone was extracted twice with 5 ml ethyl acetate in an ultrasonic bath (10 min, +25°C). The supernatants were separated and combined after centrifugation (3000 rpm, 10 min) and evaporated to dryness under nitrogen flow. The residues were dissolved in dichloromethane (50 μl; DCM) and trimethylsilylated with MSTFA (25 μl; *N*-Methyl-*N*-(trimethylsilyl) trifluoroacetamide; Pierce, Rockford, IL, USA) at 80°C for 20 min. Deglycosylation was performed with plant material into citrate-phosphate buffer (pH 5.4). After sonication (15 min), 500 μl Viscozyme L (V2010, Sigma–Aldrich, USA) was added and a heptane-layer together with internal standard was added on top of the solution. Samples were incubated at +37°C overnight and the aqueous phase was extracted with ethyl acetate as described above. Medium samples were extracted accordingly.

The samples were analyzed with an Agilent 7890A GC combined with a 5975C mass selective detector. The GC was equipped with an Agilent DB-5MS fused silica capillary column (30 m, 0.25 mm ID, phase thickness of 0.25) and the temperature program was from 70°C (1 min) to 280°C (18 min) at 10°C min^-1^. Aliquots of 1 μl were injected and the split ratio was 25:1. The data were collected over a mass range of 40–600 m/z. Identification of the compounds was based on retention times and library comparison (NIST ’08, Scientific Instrument Services, Inc., Ringoes, NJ, USA). Calibration curves of reference substances were used for quantification.

## Results

### Transient Expression of *Ri*ZS1 in *N. benthamiana*

**FIGURE 2:**
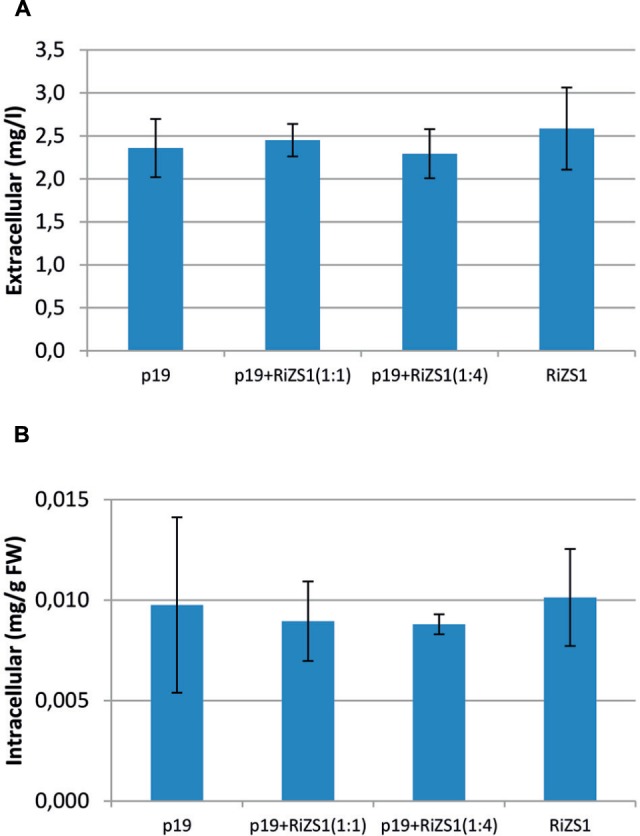
**Accumulation of raspberry ketone in *Nicotiana benthamiana* leaves after feeding with 1 mM 4-OHBA.** *Agrobacterium tumefaciens* LBA4404 carrying either p19 silencing suppressor or 35*S*-*Ri*ZS1 was infiltrated alone or in combination (in 1:1 or in 1:4 v:v ratio). **(A)** extracellular (mg/l), **(B)** intracellular (mg/g FW) levels. Error bars represent SD of biological replicates (*N* = 3) where each sample consisted of 20 infiltrated leaf disks (see Transient Expression in *N. benthamiana*).

Transient expression in *N. benthamiana* was performed using *A. tumefaciens* LBA4404 carrying either the p19 silencing suppressor (Silhavy et al., 2002) or 35*S*-*Ri*ZS1, alone or in combination. Two different dilutions of silencing suppressor p19 were tested against 35*S*:*Ri*ZS1 since the concentration required for optimal suppression activity was not known. Bioconversion of **1** was assayed after incubating leaf samples for 2 days. Product **3** accumulation was observed in all samples which received substrate **1** (**Figures 2A,B**). Only those samples without added substrate were completely devoid of **3**. However, to our surprise, control samples, i.e., samples in which only p19 was infiltrated, also contained raspberry ketone in similar amounts to those observed in *Ri*ZS1 infiltrated samples. Furthermore, p19 did not appear to have any effect on the raspberry ketone accumulation levels.

### Raspberry Ketone Production in Tobacco Hairy Roots

**FIGURE 3:**
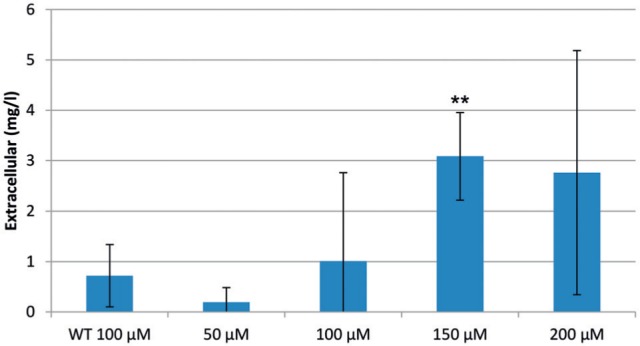
**Formation of raspberry ketone (mg/l) in the extracellular space of *N. tabacum* hairy roots expressing 35*S-Ri*ZS1 with different concentrations of fed 4-OHBA.** Feeding was performed after 9 days of cultivation and samples were taken 1 d after feeding. Error bars represent the standard deviation of three biological replicates. The experiment was repeated twice. WT, wild type. Asterisks indicate the significance level obtained by Student’s *t*-test: ^∗^*p* < 0.05, ^∗∗^*p* < 0.01, ^∗∗∗^*p* < 0.001.

The known 4-OHBA reductase *Ri*ZS1 was cloned in the Gateway^®^ plant-compatible overexpression vector and introduced to *N. tabacum* by *A. rhizogenes*. Altogether ten *N. tabacum* hairy root clones were generated carrying *Ri*ZS1 and they were subsequently screened for raspberry ketone production capacity. Hairy roots were first pre-cultivated for 9 days as described in Häkkinen et al. (2012) in order to increase the biomass as well as to reach the exponential growth phase for active intracellular metabolism. Raspberry ketone **3** was produced in three clones after feeding **1** and the best-producing clone was selected for further experiments. When hairy roots were fed with the substrate **1**, the majority of the subsequently produced **3** was secreted to the extracellular space. The accumulation of **3** was monitored for 6 days and it showed that the accumulation peak of **3** occurred already at day 1. Increased concentration of substrate resulted in an average (*N* = 3) of up to 3.0 mg/l raspberry ketone production from 150 μM **1** (**Figure 3**). A yield of up to 5.5 mg/l was obtained after feeding 200 μM, but the variation between the biological replicates was too high for reliable interpretation of the result.

In order to confirm the hypothesis drawn from the earlier *N. benthamiana* experiment, wild type (WT) hairy roots were also tested for their bioconversion capacity of **1**. We did indeed find that WT roots converted **1**–**3** at similar conversion rates as roots carrying *Ri*ZS1 (**Figure 3**). However, only three out of ten roots carrying *Ri*ZS1 produced **3** after feeding. The majority of produced **3** was secreted to the culture medium; only up to 0.2% of the whole raspberry ketone pool was found in the intracellular fraction. Fed **1** did not show spontaneous conversion into **3** during the experimental time period as tested by cell-free incubation of substrate in the medium. Furthermore, the majority of produced raspberry ketone in tobacco hairy roots was present as aglycone both in the intra- and the extracellular samples.

Hairy roots were also pre-cultivated for 21 days before feeding with **1**, in order to confirm the optimal growth phase for starting the feeding. However, raspberry ketone was not produced at all in these hairy roots, which had reached the stationary growth phase, thus confirming the necessity for rapidly dividing cells and high overall enzymatic activity during feeding.

In order to study whether elicitation could result in increased bioconversion yields in tobacco hairy roots, methyl jasmonate (MeJA) was applied simultaneously with the substrate. However, it was observed that elicitation with MeJA did not increase the amount of raspberry ketone produced (**Supplementary Table S1**).

### Bioconversion of Betuligenol and 4-OHBA by Various Plant Cell Suspensions and Hairy Root Cultures

To test the hypothesis of widely distributed plant reductase activity which is able to accept and convert raspberry ketone precursors, we screened several plant cell cultures for their bioconversion capacity. Altogether seven undifferentiated and three differentiated cell cultures from five different plant families were tested (**Table 1**). It was clearly observed that the majority of cultures were able to convert either one or both of the tested substrates into raspberry ketone. Since most of the produced **3** was found in the culture medium, the best converters were cloudberry suspension and *C. roseus* and *N. tabacum* hairy roots (**Table 1**). In this list, *C. roseus* cell suspension was the only culture which did not show conversion of either substrate.

**Table 1:** Amount of raspberry ketone produced by selected cell suspensions and hairy root cultures 1 day after feeding with 100 μM 4-OHBA or betuligenol, respectively.

Family	Species	Culture type	Raspberry ketone			
			
			4-OHBA (1)		Betuligenol (2)	
				
			Cells (μg/g DW)	Medium (mg/l)	Cells (μg/g DW)	Medium (mg/l)
*Apocynaceae*	*Catharanthus roseus*	Cell suspension	ND	ND	ND	ND
*Apocynaceae*	*Catharanthus roseus*	Hairy root	29.0	1.3	4.1	0.1
*Plumbaginaceae*	*Plumbago auriculata*	Cell suspension	0.6	ND	15.9	tr
*Poaceae*	*Hordeum vulgare*	Cell suspension	6.0	tr	tr	tr
*Rosaceae*	*Rubus arcticus*	Cell suspension	6.4	ND	tr	ND
*Rosaceae*	*Rubus chamaemorus^∗∗^*	Cell suspension	8.0	2.1	10.8	0.4
*Rosaceae*	*Rubus idaeus^∗∗^*	Cell suspension	11.1	0.1	ND	ND
*Solanaceae*	*N. tabacum* SR1	Cell suspension	2.6	0.1	tr	0.2
*Solanaceae*	*N. tabacum* SR1	Hairy root	6.0^∗^	0.7^∗^	3.0	ND
*Solanaceae*	*Hyoscyamus muticus*	Hairy root	2.0	ND	ND	ND


*ND, not detected; tr, trace, <0.05 ppm. ^∗^Mean calculated from six individual clones (2.3–12.0 ug/g DW and 0.1–1.8 mg/l). ^∗∗^freshly prepared suspensions from calli.*

After the initial screening, more detailed bioconversion studies were conducted with selected suspension cultures. Altogether six cell suspension cultures, four included in the first screening (barley, cloudberry, arctic bramble, raspberry) plus bilberry and tobacco BY-2, were subjected to further studies, including the testing of different substrate concentrations and sampling points.

Among all six cell suspensions only bilberry did not convert either **1** or **2**. Increasing levels of substrate resulted in close to dose-dependent accumulation of **3** in all other cultures tested, except for barley and arctic bramble (**Table 2**).

**Table 2:** Bioconversion of 500 μM 4-OHBA and betuligenol by plant cell suspension cultures 1 day after feeding, unless otherwise indicated.

Family	Species		Raspberry ketone				
		
		4-OHBA (1)			Betuligenol (2)		
			
		Cells (μg/g DW)	Medium (mg/l)	% in culture medium	Cells (μg/g DW)	Medium (mg/l)	% in culture medium
*Ericaceae*	*Vaccinium myrtillus*	ND	ND	0	ND	ND	0
*Poaceae*	*Hordeum vulgare^∗^*	1.0	tr	78	0.3	ND	0
*Rosaceae*	*Rubus arcticus^∗^*	12.0	ND	0	2.6	ND	0
*Rosaceae*	*Rubus chamaemorus*	29.1	0.6	75	20.5	1.1	88
*Rosaceae*	*Rubus idaeus*	80.2	1.9	81	2.2	0.2	94
*Solanaceae*	*N. tabacum* BY-2	158.2	7.4	75	106.3	5.0	75


*^∗^sample taken after 5 days; ND, not detected; tr, trace, <0.05 ppm.*

The three tested *Rubus* species showed rather different patterns of accumulation of **3**. Cloudberry converted both **1** and **2** at almost equal rates; raspberry showed a preference for **1** and arctic bramble was able to convert both substrates; however, the product accumulated only in the intracellular space (**Table 2**). Based on the earlier reports of a hydrogen acceptor requirement during betuligenol bioconversion, acetone was added to the incubation mixture of hairy roots used in this study. However, this did not result in increased production of **3** (**Supplementary Table S2**). Among the cultures tested in this study, *C. roseus* and *V. myrtillus* cell suspension cultures did not produce **3** (**Tables 1** and **2**).

By far the best conversion of both **1** and **2**–**3** was achieved with *N. tabacum* BY-2, raspberry and cloudberry cells (**Table 2**), with tobacco BY-2 showing up to 12% total conversion rate. All the cultures tested, except arctic bramble, accumulated more than 75% of the product in the culture medium (**Table 2**).

**FIGURE 4:**
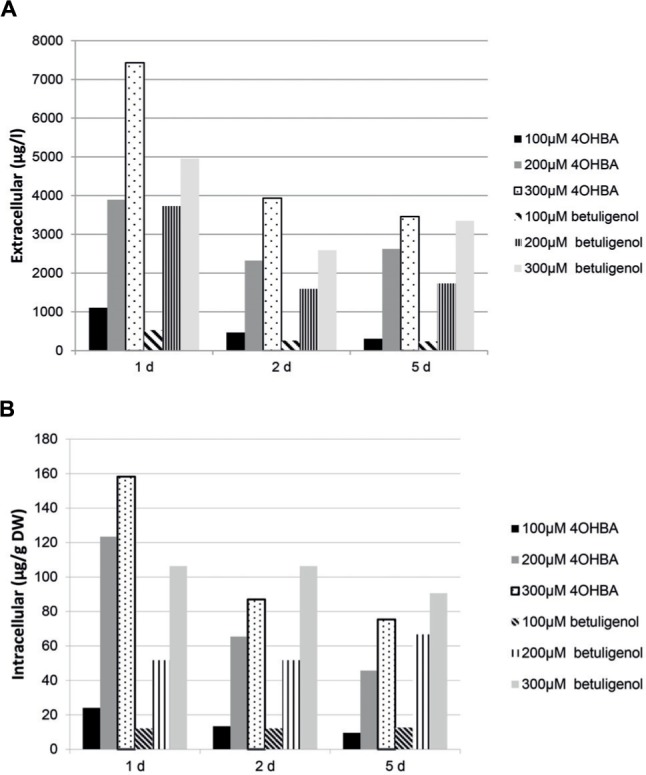
**Accumulation of raspberry ketone in culture medium (μg/l) **(A)** and in intracellular space (μg/g DW) **(B)** in *N. tabacum* BY-2 cell suspension culture after feeding with either 4-OHBA or betuligenol**.

The temporal accumulation pattern observed for *N. tabacum* BY-2 (**Figure 4**) shows that the highest amount of **3** accumulated both in the intra- and extracellular space 1 day after substrate feeding. After 1 day the level of **3** declined, possibly due to degradation or further metabolism. In the case of barley and arctic bramble the highest accumulation of **3** occurred only after 5 days. Tyrosol, a suggested metabolite resulting from degradation of **3**, was not detected in cell culture samples tested in this study, based on the mass fragmentation patterns reported by Angerosa et al. (1995).

## Discussion

### Tobacco as a Production Platform for Raspberry Ketone

As expected, *N. benthamiana* did not accumulate **3** without added substrate, since tobacco is not known to possess the whole pathway leading to raspberry ketone. However, to our surprise, **3** also accumulated in p19 infiltrated control samples in amounts similar to those in *Ri*ZS1 infiltrated samples. Similarly, in *N. tabacum* hairy root cultures, extracellular concentration of converted **3** was similar in WT hairy roots (2.8 mg/l) and hairy roots carrying *Ri*ZS1 (3.0 mg/l) (**Figure 3**; **Table 1**). *Ri*ZS1 belongs to the medium chain reductase/dehydrogenase (MDR)/zinc-dependent ADH–like family of proteins, a diverse group of proteins related to class I mammalian ADH. MDR proteins constitute a large enzyme superfamily with close to 1000 members (Nordling et al., 2002), and display a wide variety of activities including ADH, quinone reductase, cinnamyl reductase and numerous others. It is interesting to note that the protein sequence of *Ri*ZS1 exhibits 76% similarity and 74% nucleotide sequence similarity with *N. tabacum* allyl ADH (DDBJ/EMBL/GenBank accession nr. AB036735) (**Supplementary Figure S1**).

This tobacco gene was later renamed as *Nt*DBR (*N. tabacum* Double Bond Reductase) by Mansell et al. (2013), making it a prime candidate responsible for the observed activities in WT *N. benthamiana* and *N. tabacum* hairy roots. *Nt*DBR has 70% homology to the NADPH-dependent oxidoreductases belonging to a plant ζ-crystallin family [leukotriene B4 dehydrogenase family (LTD), a subfamily of the MDR superfamily proteins] and it catalyzes a reversible dehydrogenation of allylic alcohols or ketones (Hirata et al., 2000). Both *Ri*ZS1 and *Nt*DBR belong to the LTD family and both have an NADPH co-factor binding site AXXGXXG.

The accumulation peak of **3** in tobacco hairy roots and cell suspensions, as well as in most other cultures tested, was observed already at day 1 (**Figure 4**). This is in accordance with the results obtained with *A. belladonna* by Srivastava et al. (2013), i.e., bioconversion taking place already at day 1 although the maximum conversion was obtained at day 5. Since only three out of ten roots carrying *Ri*ZS1 actually produced **3** after feeding, it is suspected that co-supression (Vaucheret and Fagard, 2001) occurred in the remaining transgenic clones, resulting most probably from a high sequence similarity between *Ri*ZS1 and corresponding endogenous reductases (**Supplementary Figure S1**). Production levels up to 5.5 mg/l of **3** were recorded for hairy roots after feeding 200 μM of **1**. These levels correspond to those reported by Beekwilder et al. (2007) with 5 mg/l production rates obtained by *E. coli* expressing chalcone synthase from *Rubus idaeus*. In fresh raspberry fruits, levels of 0.01–0.17 μg/g have been reported (Hrazdina and Zheng, 2006). Accumulation levels up to 20 μg/g have been obtained in elicited raspberry cell cultures (Hrazdina and Zheng, 2006).

Glycosylation is a very important detoxification mechanism in xenobiotic metabolism (Schmidt et al., 2006; Häkkinen et al., 2012). In addition, glycosylation facilitates the conversion of water-insoluble substances into water-soluble compounds and may also be needed in aiding the transport of the particular compound to e.g., vacuole or apoplastic space. Particularly, glycosylation by cultured plant cells has been the subject of increasing attention, since one-step enzymatic glycosylations by plant cells are more convenient than chemical glycosylations, which require tedious steps of protection and deprotection of the sugar hydroxyl groups. For this reason possible glycosylated conjugates of raspberry ketone were screened following enzyme-assisted deglycosylation. However, the main part of **3** was present as aglycone in tobacco hairy roots. Glycosylation of fed raspberry ketone was earlier demonstrated by Shimoda et al. (2007) with cultured cells of *Phytolacca americana*, which converted fed raspberry ketone into β-glycosides after hydroxylation during a 3 day incubation period.

Altogether, the conversion efficiency of hairy roots was much higher than that of infiltrated leaves, 12% versus 2%, respectively. The difference is even more striking if the incubation time is taken into account, since hairy roots were sampled after 1 day and infiltrated leaves after 2 days. It is noteworthy that the bioconversion yield obtained in this study with *N. tabacum* hairy roots is higher than the bioconversion of betuligenol reported by Srivastava et al. (2013), who obtained an overall 0.5% bioconversion rate at day 1 and a maximum of 7% bioconversion after 5 days with *A. belladonna* hairy roots (calculated from the presented data). The microbial bioconversion capacity of *E. coli* converting **1**–**3** was reported as 40% after 1 day by Beekwilder et al. (2007), whereas bioconversion of **2**–**3** using *Rhodococcus* cells in buffer with 10% (v/v) acetone was as high as 89% after 2 days (Kosjek et al., 2003). As a conclusion, although unable to compete with microbial bioconversion systems in terms of yield, *N. tabacum* hairy roots offer an efficient plant-based bioconversion platform for production of natural raspberry ketone.

### Betuligenol and 4-OHBA are Converted to Raspberry Ketone by Various Plant Species

#### Hairy Roots

Bioconversion capacity differed even between closely related plant species, e.g., the two Solanaceae. Whereas *N. tabacum* hairy roots were able to convert betuligenol, the related *H. muticus* did not show bioconversion. Earlier, Srivastava et al. (2013) reported that hairy roots of another Solanaceae, *A. belladonna*, were able to convert **2** to raspberry ketone. Since Kosjek et al. (2003) had reported from work with bacterial cultures that **2** would require acetone as a hydrogen acceptor, hairy roots were fed with betuligenol together with acetone at a concentration of 1% (v/v). However, the efficiency of bioconversion was not improved by acetone addition. This is in accordance with reported betuligenol bioconversion in *A. belladonna*, which performed the bioconversion as such (Srivastava et al., 2013). This difference between prokaryotic and plant platforms may be explained by the differences in oxidative enzymes. Plants possess a huge variety of cytochrome P450 enzymes together with an abundant hydrogen acceptor pool, carrying out diverse oxidative reactions, whereas the number of P450 enzymes in prokaryotes is generally much lower, and *E. coli* does not possess any at all (Werck-Reichhart and Feyereisen, 2000).

#### Cell Suspensions

Tobacco BY-2 performed best in this study, with 12% total conversion rate, which is higher than the earlier reported plant-based conversion by *A. belladonna* hairy roots (7%, Srivastava et al., 2013). *Rubus* sp., namely raspberry and cloudberry constitute further potent plant platforms (**Table 2**). The cell density of highly multiplying BY-2 culture is high during the exponential growth phase. However, the total amount of biomass at the time of sampling in tobacco BY-2 was approximately twofold compared to other cultures tested. Thus, the number of cells performing the bioconversion cannot be the only reason for the high bioconversion rates in tobacco BY-2. It should also be noted that different cell cultures might possess different optimal growth stages for specific bioconversions, and thus accurate comparisons of the bioconversion potential of different cultures cannot be made without more detailed studies.

Accumulation in culture medium, as seen in all the cultures tested except for arctic bramble, is a highly appreciated phenomenon compared to the typical intracellular location of secondary metabolites. Although product degradation may occur as a function of time (**Figure 4**), by timing the product recovery correctly the yields of produced **3** remain rather high. Earlier it had been suggested that raspberry ketone is degraded into tyrosol in fungi (Fuganti et al., 1996), but tyrosol was not detected in our cell culture samples.

The demand for “natural” raspberry ketone is growing considerably, partly due to the recent findings related to its favorable properties related to weight regulation and skin-lightening (Morimoto et al., 2005; Park, 2010; Lin et al., 2011). Bioconversion is an efficient and ‘green’ technology to convert various substrates into more valuable or less toxic compounds. In this work we have shown that a wide variety of plant cell cultures can be utilized for bioconversion purposes, allowing production in a contained environment, independent of environmental conditions and free of pesticides and contaminants. In the case of raspberry ketone, a very high-value natural flavor substance can be produced in plant cell cultures by applying 4-hydroxybenzalacetone or betuligenol, both of which are rather cheap and readily available precursors. Accumulation in the extracellular space, as shown in this study, is beneficial for compound recovery. Downstream processing may account for as much as 80% of overall production costs and for this reason less complex compound isolation and purification from the culture medium has a major impact on the total costs of a biotechnological process.

## Author Contributions

SH, TS-L, K-MO-C, HR designed the research; SH and TS-L performed the research; SH, TS-L, HR analyzed data; SH, HR wrote the paper.

## Conflict of Interest Statement

The authors declare that the research was conducted in the absence of any commercial or financial relationships that could be construed as a potential conflict of interest.
