# Supplementary material for: Bioconversion to Raspberry Ketone is Achieved by Several Non-related Plant Cell Cultures
Source: Front Plant Sci. 2015 Nov 24;6:1035. doi: 10.3389/fpls.2015.01035 (PMC4656793; doi:10.3389/fpls.2015.01035)
Supplement: Supplementary file 1 [file Data_Sheet_1.PDF]

Supplementary Material

Bioconversion to raspberry ketone is achieved by several non-related plant cell cultures

Suvi T. Häkkinen, Tuulikki Seppänen-Laakso, Kirsi-Marja Oksman-Caldentey, Heiko Rischer*

*** Correspondence:** Heiko Rischer: heiko.rischer@vtt.fi

Supplementary Fig S1.

```
NtADH          ----MAEEVSNKQVILKNYVTGYPKESDMEIKNVTIKLKVPEGSNDVVVKNLYLSCDPYM
RiZS1          MASGGEMQVSNKQVIFRDYVTGFPKESDMELTTRSITLKLPQGSTGLLLKNLYLSCDPYM
                      :*******:::****:*******:.. :*.**:*:**. :::***********

NtADH          RSRMRKI-EGSYVESFAPGSPITGYGVAKVLESGDPKFQKGDLVWGMTGWEEYSIITPTQ
RiZS1          RARMTNHHRLSYVDSFKPGSPIIGYGVARVLESGNPKFNPGDLVWGFTGWEEYSVITATE
               *:** :  . ***:** ***** *****:*****:***: ******:*******:** *:

NtADH          TLFKIHDKDVPLSYYTGILGMPGMTAYAGFHEVCSPKKGETVFVSAASGAVGQLVGQFAK
RiZS1          SLFKIHNTDVPLSYYTGLLGMPGMTAYAGFYEICSPKKGETVYVSAASGAVGQLVGQFAK
               :*****:.*********:************:*:*********:*****************

NtADH          MLGCYVVGSAGSKEKVDLLKSKFGFDEAFNYKEEQDLSAALKRYFPDGIDIYFENVGGKM
RiZS1          LTGCYVVGSAGSKEKVDLLKNKFGFDEAFNYKEEADLDAALRRYFPDGIDIYFENVGGKM
               : ******************.************* **.***:******************

NtADH          LDAVLVNMKLYGRIAVCGMISQYNLEQTEGVHNLFCLITKRIRMEGFLVFDYYHLYPKYL
RiZS1          LDAVLPNMRPKGRIAVCGMISQYNLEQPEGVRNLMALIVKQVRMEGFMVFSYYHLYGKFL
               ***** **:  **************** ***:**:.**.*::*****:**.***** *:*

NtADH          EMVIPQIKAGKVVYVEDVAHGLESAPTALVGLFSGRNIGKQVVMVSRE
RiZS1          ETVLPYIKQGKITYVEDVVDGLDNAPAALIGLYSGRNVGKQVVVVSRE
               * *:* ** **:.*****..**:.**:**:**:****:*****:****
```

Supplementary Fig S1. Amino acid alignment of *Nt*DBR (NCBI Accession nr AB036735) and *Ri*ZS1 (NCBI Accession nr JN166691.1). Sequence alignment was generated using the CLUSTAL W omega multiple sequence alignment program (Thompson et al. 1994). NADPH-binding domain is underlined.

Supplementary Material

Bioconversion to raspberry ketone is achieved by several non-related plant cell cultures

Suvi T. Häkkinen, Tuulikki Seppänen-Laakso, Kirsi-Marja Oksman-Caldentey, Heiko Rischer*

*** Correspondence:** Heiko Rischer: heiko.rischer@vtt.fi

Supplementary Fig S2.

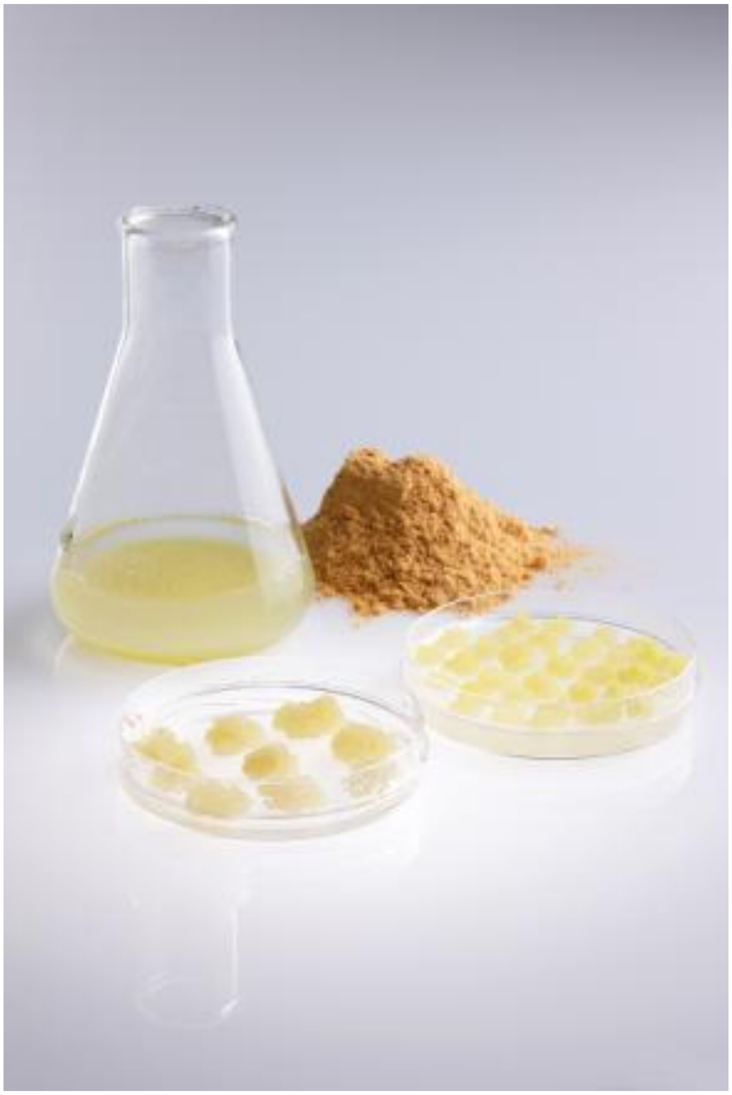

Supplementary Fig S2. *Rubus chamaemorus* callus and cell suspension cultures

Bioconversion to raspberry ketone is achieved by several non-related plant cell cultures

Suvi T. Häkkinen, Tuulikki Seppänen-Laakso, Kirsi-Marja Oksman-Caldentey, Heiko Rischer*

*** Correspondence:** Heiko Rischer: heiko.rischer@vtt.fi

Supplementary Fig S3.

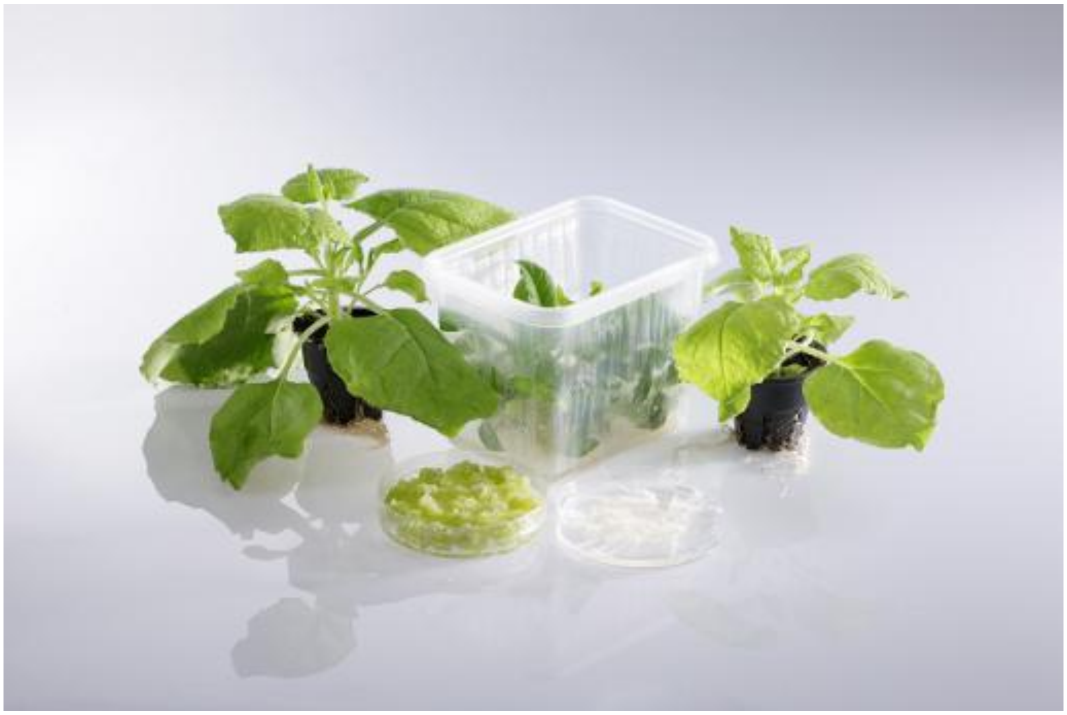

Supplementary Fig S3. *Nicotiana* plants, callus and hairy root cultures

Bioconversion to raspberry ketone is achieved by several non-related plant cell cultures

Suvi T. Häkkinen, Tuulikki Seppänen-Laakso, Kirsi-Marja Oksman-Caldentey, Heiko Rischer*

*** Correspondence:** Heiko Rischer: heiko.rischer@vtt.fi

Supplementary Fig S4.

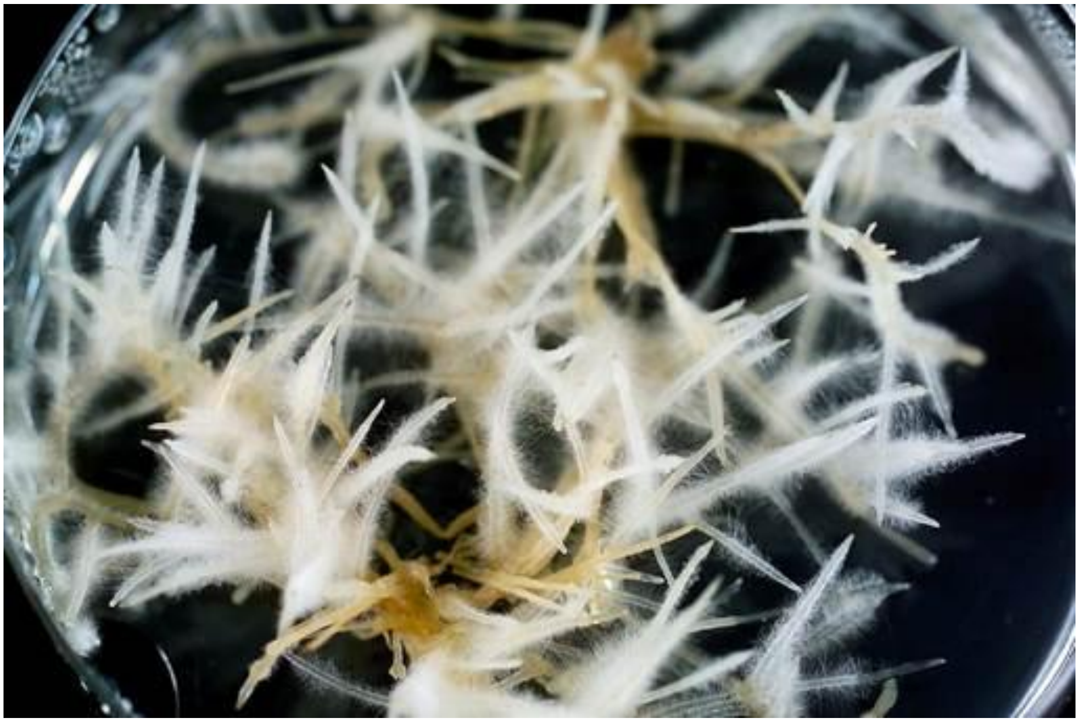

Supplementary Fig S4. *Nicotiana* hairy roots

Supplementary Material

Bioconversion to raspberry ketone is achieved by several non-related plant cell cultures

Suvi T. Häkkinen, Tuulikki Seppänen-Laakso, Kirsi-Marja Oksman-Caldentey, Heiko Rischer*

*** Correspondence:** Heiko Rischer: heiko.rischer@vtt.fi

Supplementary Fig S5.

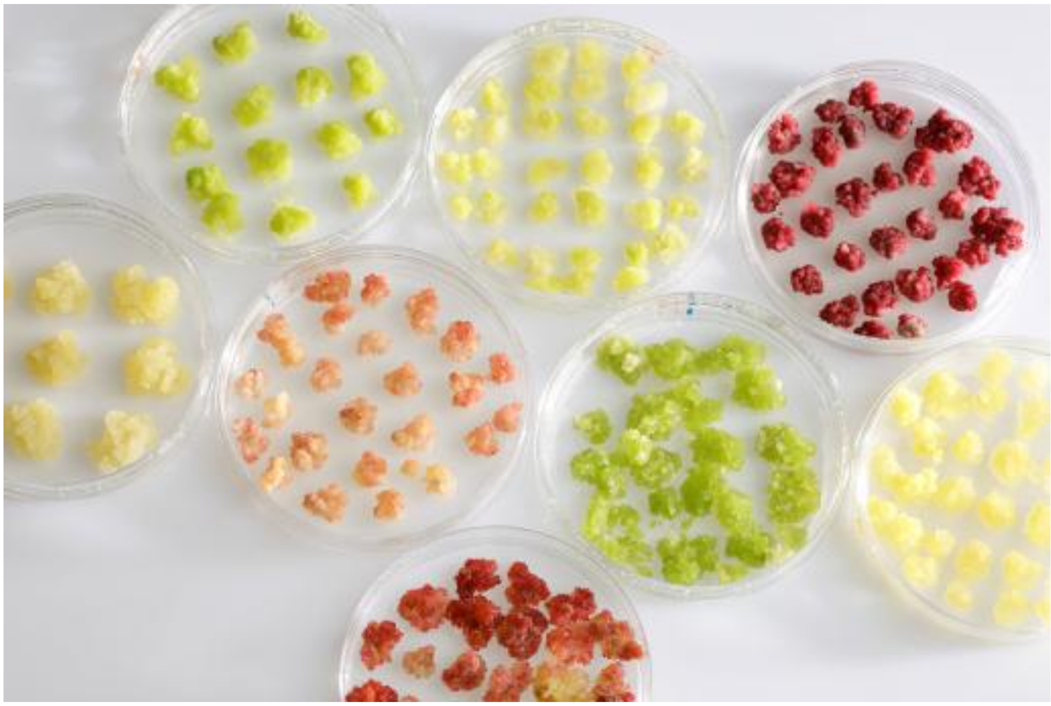

Supplementary Fig S5. Various callus cultures

Supplementary Material

Bioconversion to raspberry ketone is achieved by several non-related plant cell cultures

Suvi T. Häkkinen, Tuulikki Seppänen-Laakso, Kirsi-Marja Oksman-Caldentey, Heiko Rischer*

*** Correspondence: Heiko Rischer**: heiko.rischer@vtt.fi

Supplementary Table S1.

Accumulation of raspberry ketone in tobacco hairy roots carrying *Ri*ZS1 after feeding 4-OHBA (50 µM) and methyl jasmonate (MeJA) (50 µM). Samples were taken at day 2 after feeding.

	**intracellular (µg/g DW)**		**culture medium (mg/l)**	
	**4-OHBA**	**4-OHBA+MeJA**	**4-OHBA**	**4-OHBA+MeJA**
**Clone 1**	0.24	0.22	0.21	0.08
**Clone 2**	0.28	0.20	tr	tr

Supplementary Material

Bioconversion to raspberry ketone is achieved by several non-related plant cell cultures

Suvi T. Häkkinen, Tuulikki Seppänen-Laakso, Kirsi-Marja Oksman-Caldentey, Heiko Rischer*

*** Correspondence:** Heiko Rischer: heiko.rischer@vtt.fi

Supplementary Table S2.

Accumulation of raspberry ketone in hairy roots of three species after feeding betuligenol (100 µM) and acetone (1 % v/v). Samples were taken after 1 day. tr: trace amounts, ND: not detected.

	**intracellular (µg/g DW)**		**culture medium (mg/l)**	
	**Betuligenol**	**Betuligenol + acetone**	**Betuligenol**	**Betuligenol + acetone**
***N. tabacum* SR1**	0.003	tr	ND	ND
H. muticus	ND	ND	ND	ND
C. roseus	0.004	0.003	0.123	0.127
